# Rectal Bleeding Associated With Chronic Pancreatitis

**DOI:** 10.1155/1991/85468

**Published:** 1991

**Authors:** Ch. Seiler, G. Fielding, L. H. Blumgart, J. Triller, H. R. Schultheiss

**Affiliations:** 1 University Clinic of Visceral and Transplantation Surgery Inselspital Bern Bern CH-3010 Switzerland; 2 Radiological Department Inselspital Bern Switzerland; 3 Department of Surgery District Hospital of Biel Switzerland

## Abstract

Pseudocyst formation, with its attendant complications of compression, rupture, bleeding and fistula formation, is a well known complication of chronic pancreatitis. In 1966 Berne and Edmondson drew attention to the often fatal outcome of pancreatico-colonic fistula complicated by hemorrhage. We present two cases of this rare complication of chronic pancreatitis as defined by the Marseille classification.


*HPB Surgery*, 1991, Vol. 3, pp. 199-203
Reprints available directly from the publisher
Photocopying permitted by license only

© 1991 Harwood Academic Publishers GmbH
Printed in the United Kingdom

### CASE REPORT

### RECTAL BLEEDING ASSOCIATED WITH CHRONIC PANCREATITIS

Ch. SEILER., G. FIELDING and L.H. BLUMGART
*University Clinic of Visceral and Transplantation Surgery, Inselspital Bern, CH-3010 Bern, Switzerland*

J. TRILLER
*Radiological Department, Inselspital Bern*

H.R. SCHULTHEISS
*Department of Surgery, District Hospital of Biel*

*(Received 10 April 1990)*

Pseudocyst formation, with its attendant complications of compression, rupture, bleeding and fistula formation, is a well known complication of chronic pancreatitis. In 1966 Berne and Edmondson drew attention to the often fatal outcome of pancreatico-colonic fistula complicated by hemorrhage[3]. We present two cases of this rare complication of chronic pancreatitis as defined by the Marseille classification[1,2].

### CASE REPORTS

*Case 1:* A 62 year old man with a long history of recurrent pancreatitis and a known pseudocyst in the head of the pancreas was admitted to hospital as an emergency with rectal bleeding. He had recently undergone coronary artery bypass grafting and was taking the thrombocyte aggregation blocker (Calciumacetylosalicylicum). On admission the pulse rate was 96/min, the blood pressure 80/50 mmHg. Haemoglobin was 8.9 g%. He was resuscitated with intravenous fluid.

An ultrasound of the abdomen showed a 5 × 5 cm rounded lesion in the pancreatic head with an inhomogeneous echostructure. A computerized axial tomographic scan showed a pseudocyst in the tail of the pancreas with inhomogeneous shadowing and blood-equivalent density values. Gas was present in the cyst. There was intensive inflammatory change around the pancreas and left colonic flexure. Coeliac angiography showed an elongated splenic artery with a 2 × 2 cm false aneurysm at the splenic hilus. Colonoscopy showed a granulated fistula at the left colonic flexure.

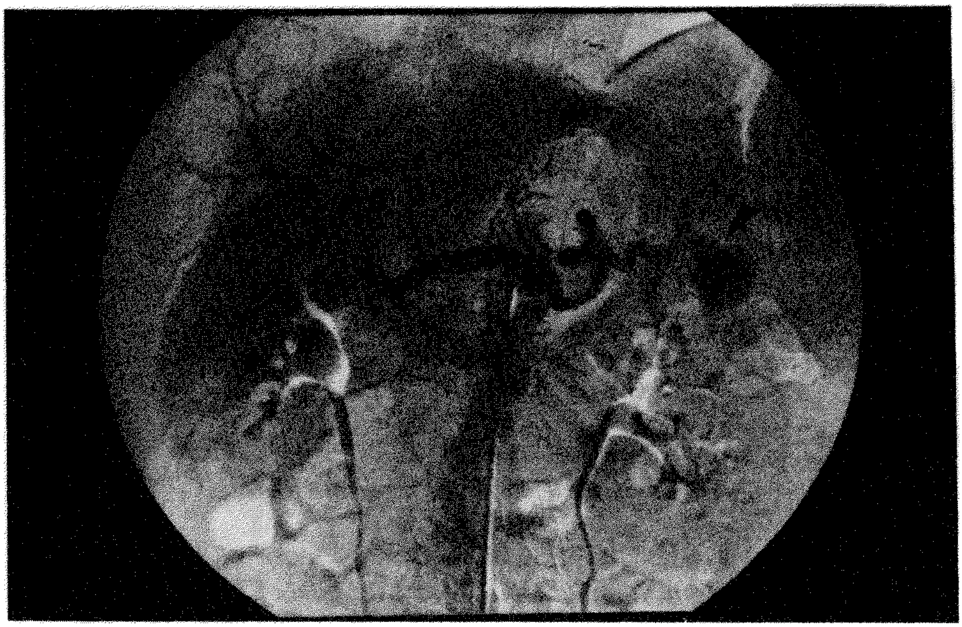

**Figure 1** *Angiography*. Angiography showing a 2 × 2 cm false aneurysm of the splenic artery (arrow) overlying the pancreatic tail.

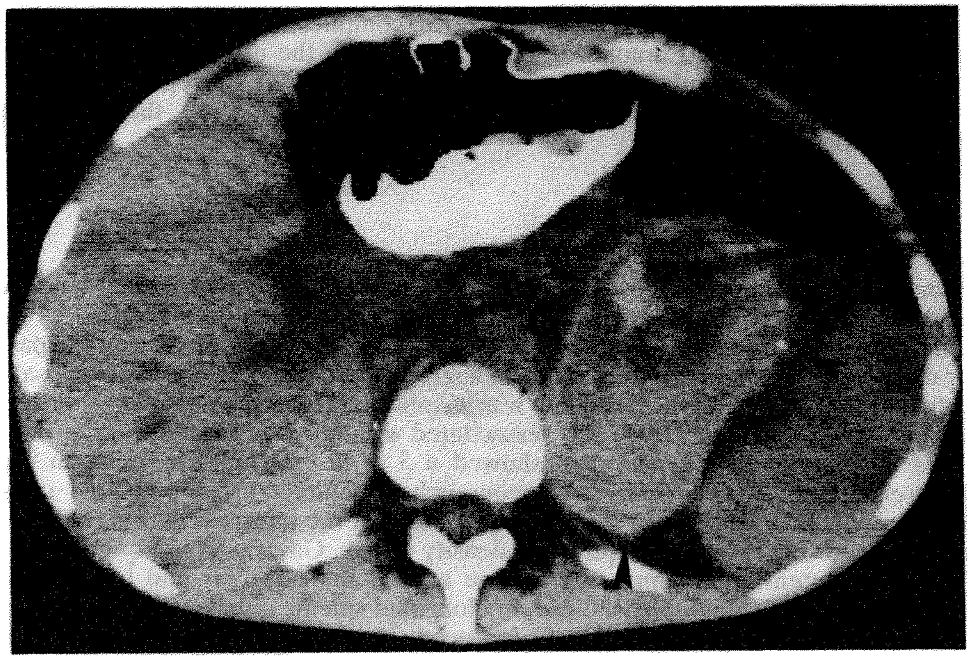

**Figure 2** *CAT-Scan*. 10 × 5 cm pancreatic pseudocyst with calcification in the cyst membrane (arrow). Density measurement of the contents is equivalent to blood.

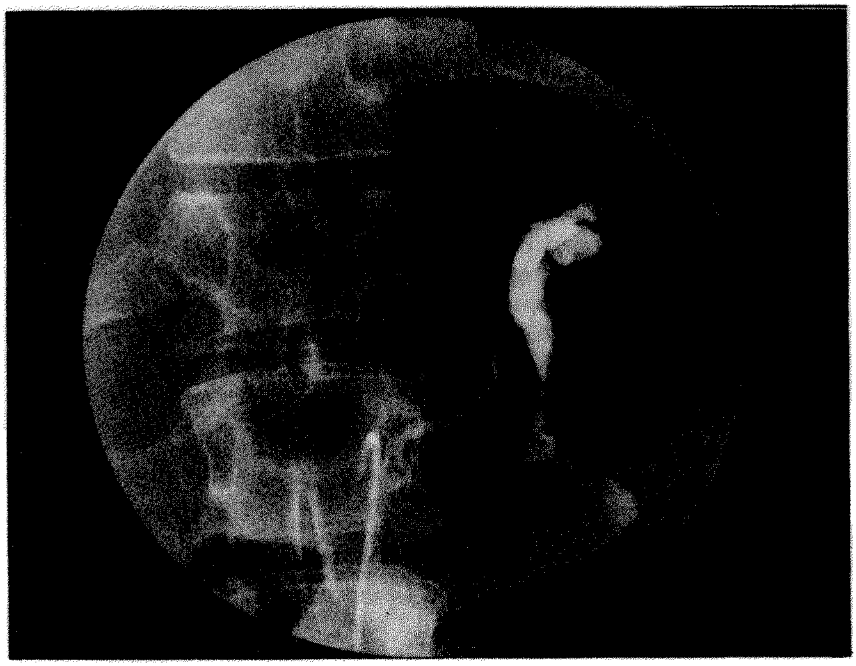

**Figure 3** *Angiography*. Superselective demonstration of splenic artery. The proximal part of the vessel is spastic in the region of neck/tail of the pancreas and demonstrates a contrast medium extravasation into a pseudocyst.

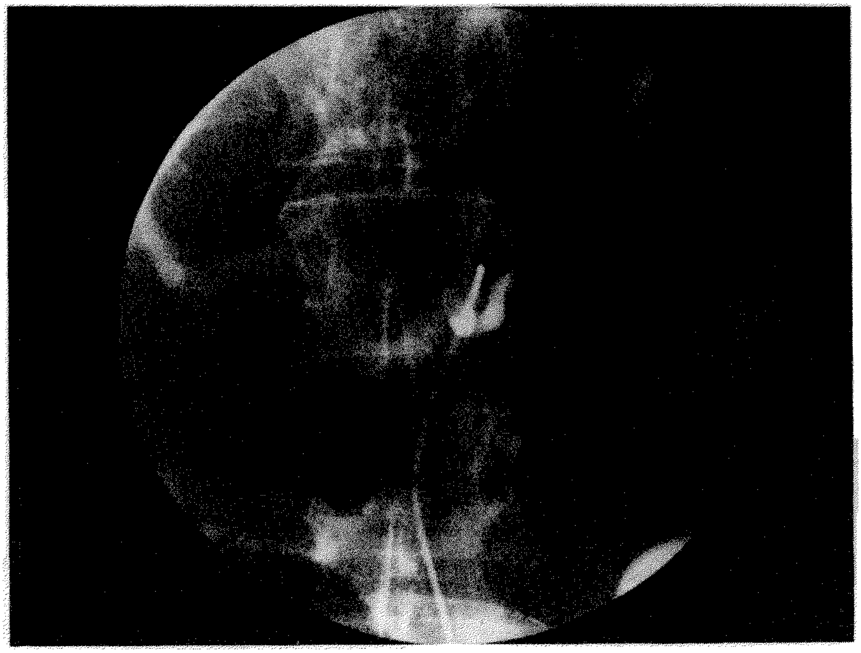

**Figure 4** Embolisation of the splenic artery with ivalon particles. No further extravasation into pseudocyst visable. However, contrast medium is now seen in the hepatic artery.

Laparotomy confirmed the preoperative diagnosis of bleeding into the colon through a pseudocyst colonic-fistula. Distal pancreatectomy, splenectomy and colonic resection en bloc were performed. The patient made an uneventful recovery.

*Case 2:* A 29 year old alcoholic heroin addict had first presented with alcohol induced pancreatitis in 1983 with severe pain and a pancreatic pseudocyst. In 1986 he suffered thrombosis of the superior vena cava subsequent to subclavian catheterization at the time of planned pancreatic resection and was anticoagulated. His course subsequently was uneventful until 2 weeks prior to admission, when he developed loss of apetite, abdominal pain and constipation. He presented with severe melaena, pulse rate was 136 min., blood pressure 70/50. Haemoglobin was 4.9 g/%. After resuscitation CAT-scan showed a large (10 × 5 cm) partly calcified pseudocyst, with inhomogeneous content suggesting hemorrhage. Splenic artery angiography showed narrowing of the lumen and rupture of the mid-part of the vessel into the pseudocyst with active bleeding in progress (Figure 3). The splenic artery was successfully embolized. Colonoscopy showed a 5 × 5 cm pseudocystocolic fistula in the left colonic flexure and allowed a free view into the pancreatic pseudocyst (Figure 5).

Subsequent treatment was conservative and the patient recovered to be discharged 6 weeks later. Repeated CT-scan 10 months after embolisation showed no residual cyst and colonoscopy was normal.

**Figure 5** *Colonoscopy*. Huge pseudocysto-colic fistula in the region of left colon flexure. View into the pseudocyst, showing necrosis and blood clot. (see colour plate at back of issue)

### DISCUSSION

It is well known that pancreatic pseudocysts are prone to the complications of hemorrhage, infection rupture and compression of surrounding organs especially of the stomach, bile duct and colon. Organ compression and fistula formation are often asymptomatic. However, when a fistula is complicated by haemorrhage the outcome is frequently fatal[4]. The two cases reported highlight the sudden overwhelming nature of hemorrhage which can occur from the splenic artery. With modern radiology prior deliniation of the cause of bleeding is possible allowing planning of the appropriate procedure for each patient[6].

Until recently only operative intervention and suture control of the bleeding vessel with concomitant resection of colon and pancreas held any real chance of success. Interventional radiological techniques now allow control of bleeding[7,8]. Some pseudocystocolic fistula may be asymptomatic, once bleeding is controlled there is no pressing need to deal surgically with the fistula. This is well illustrated by the second case where spontaneous closure of the fistula occurred.

Melaena from bleeding pseudocystocolic fistula is rare but must be remembered in patients with chronic pancreatitis. Embolisation of the bleeding vessel, if successful, may allow spontaneous resolution of the cystocolic fistula. In severely ill and compromised patients this offers a real advantage over operation.

### *References*

1. Gyr, K., v. Singer, M. and Sarles, H. (1984) Revidierte Marseille - Klassifikation der Pankretitis Second International Symposium on the Classification of Pancreatitis, Marseille Schweiz. Aerztezeitung, Band 65, 46, 14.11.1984
2. Sarner, M. and Cotton, P.B. Classification of Pancreatitis International Workshop, King's College Cambridge. *Gut*, **84**, 25, 756–59
3. Berne, T.V. and Edmondson, H.A. (1966) Colonic fistulization due to pancreatitis, *Am. J. Surg.* **111**, 359-363
4. Stanley, J.C., Frey, C.F., Miller, T.A., Lindenauer, S.M. and Child, C.G. (1976) Major arterial hemorrhage; a complication of pancreatic pseudocysts and chronic pancreatitis, *Arch. Surg.* **111**, 435–440
5. Riviluoto, T., Schröder, T., Kivilaakso, E. and Lempinen, M. (1984) Acute haemorrhage associated with pancreatic pseudocyst and chronic pancreatitis, *Ann. Chir. et Gynaecol.* **73**, 214–18
6. Walter, J.F., Chuang, V.P., Bookstein, J.J., Reuter, S.R., Cho, K.J. and Pulmano, C.M. (1977) Angiography of massive hemorrhage secondary to pancreatic disease. *Diag. Radiol.* **124**, 337–42
7. Vujic, I., Andersen, B.L., Stanley, J.H. and Gobien, R.P. (1984) Pancreatic and peripancreatic vessels, embolization for control of bleeding in pancreatitis. *Radiology*, **150**, 51–55
8. Smith, J.A., Maclenst, D.G. and Collier, N.A. (1989) Aneurysm of the visceral arteries. *Aust. NZ J. Surg.* **59**, 329–34

*(Accepted by S. Bengmark 10 April 1990)*

